# *RNF213* Rare Variants in Slovakian and Czech Moyamoya Disease Patients

**DOI:** 10.1371/journal.pone.0164759

**Published:** 2016-10-13

**Authors:** Hatasu Kobayashi, Miroslav Brozman, Kateřina Kyselová, Daša Viszlayová, Takaaki Morimoto, Martin Roubec, David Školoudík, Andrea Petrovičová, Dominik Juskanič, Jozef Strauss, Marián Halaj, Peter Kurray, Marián Hranai, Kouji H. Harada, Sumiko Inoue, Yukako Yoshida, Toshiyuki Habu, Roman Herzig, Shohab Youssefian, Akio Koizumi

**Affiliations:** 1 Department of Health and Environmental Sciences, Kyoto University Graduate School of Medicine, Kyoto, 6068501 Japan; 2 Department of Neurology, Faculty Hospital Nitra, Constantine Philosopher University, Nitra, 94901 Slovakia; 3 Center of Medical Genetics, Frýdek-Místek, 370 08 Czech Republic; 4 Department of Neurosurgery, Kyoto University Graduate School of Medicine, Kyoto, 6068507 Japan; 5 Department of Neurology, Comprehensive Stroke Center, Ostrava University Faculty of Medicine and University Hospital, Ostrava-Poruba, 708 52 Czech Republic; 6 Center for Research and Science, Department of Nursing, Faculty of Health Science, Palacký University, Olomouc, 77515 Czech Republic; 7 Department of Radiology, Faculty Hospital Nitra, Constantine Philosopher University, Nitra, 94901 Slovakia; 8 Cardio Center, Faculty Hospital Nitra, Constantine Philosopher University, Nitra, 94901 Slovakia; 9 Laboratory of Nutritional Sciences, Department of Food Science and Nutrition, Mukogawa Women’s University, Nishinomiya, 6638121 Japan; 10 Department of Neurology, Comprehensive Stroke Center, Charles University Faculty of Medicine and University Hospital, Hradec Králové, 500 38 Czech Republic; 11 Laboratory of Molecular Biosciences, Graduate School of Medicine, Kyoto University, Kyoto, 6068501 Japan; Huashan Hospital Fudan University, CHINA

## Abstract

*RNF213/Mysterin* has been identified as a susceptibility gene for moyamoya disease, a cerebrovascular disease characterized by occlusive lesions in the circle of Willis. The p.R4810K (rs112735431) variant is a founder polymorphism that is strongly associated with moyamoya disease in East Asia. Many non-p.R4810K rare variants of *RNF213* have been identified in white moyamoya disease patients, although the ethnic mutations have not been investigated in this population. In the present study, we screened for *RNF213* variants in 19 Slovakian and Czech moyamoya disease patients. A total of 69 *RNF213* coding exons were directly sequenced in 18 probands and one relative who suffered from moyamoya disease in Slovakia and the Czech Republic. We previously reported one proband harboring *RNF213* p.D4013N. Results from the present study identified four rare variants other than p.D4013N (p.R4019C, p.E4042K, p.V4146A, and p.W4677L) in four of the patients. P.V4146A was determined to be a novel *de novo* mutation, and p.R4019C and p.E4042K were identified as double mutations inherited on the same allele. P.W4677L, found in two moyamoya disease patients and an unaffected subject in the same pedigree, was a rare single nucleotide polymorphism. Functional analysis showed that *RNF213* p.D4013N, p.R4019C and p.V4146A-transfected human umbilical vein endothelial cells displayed significant lowered migration, and *RNF213* p.V4146A significantly reduced tube formation, indicating that these are disease-causing mutations. Results from the present study identified *RNF213* rare variants in 22.2% (4/18 probands) of Slovakian and Czech moyamoya disease patients, confirming that *RNF213* may also be a major causative gene in a relative large population of white patients.

## Introduction

Moyamoya disease (MMD) is a progressive cerebrovascular disease characterized by bilateral stenoses of the arteries around the circle of Willis with prominent arterial collateral circulation [1–3]. Recently, *RNF213/Mysterin* was identified as a susceptibility gene for MMD, and its p.R4810K variant (rs112735431) has been shown to be a founder polymorphism that is strongly associated with MMD in East Asia [4,5]. Many *RNF213* rare variants other than p.R4810K have been identified in MMD patients in ethnically diverse populations, including Asians, whites, and Hispanics, while p.R4810K is absent in non-Asian populations [6]. These reports highlight the importance of screening for *RNF213* rare variants in MMD patients.

In present study, we screened *RNF213* rare variants in 19 white Slovakian and Czech MMD patients. Results revealed four rare variants, including a novel *de novo* mutation and a haplotype carrying two mutations.

## Materials and Methods

### Patients

Eighteen Slovakian or Czech probands and one relative with MMD (Table 1) were recruited for this study from 2008 to 2015. Among them, one proband harboring *RNF213* p.D4013N was previously reported by our group [4]. This study was approved by the Institutional Review Board and Ethics Committee of Kyoto University School of Medicine, Japan (Approval number: G342; approval date: 12/25/2009) and the Ethics Committee of the University Hospital Olomouc and Palacký University Faculty of Medicine and Dentistry in Olomouc, Czech Republic (Approval number: 62/10; approval date: 8/18/2008).

**Table 1:** Clinical characteristics of Slovak and Czech probands with MMD.

Proband No.	ID in present paper	Gender	Age (year)	Onset age (year)	Onset type	Laterality	RNF213 rare variant	Reference
1		Male	44	30	CI	Bilateral	p.D4013N	[4]*
2	II-2 Family 1	Female	21	9	TIA	Bilateral	p.V4146A	
3	II-1 Family 2	Female	28	19	CI	Bilateral	p.R4019C, p.E4042K	
4	III-2 Family 3	Female	27	5	paresis	Bilateral	p.W4677L	
mother of 4	II-2 Family 3	Female	48	31	CI	Unilateral	p.W4677L	
5		Female	34	30	CI	Bilateral	(-)	
6		Female	41	18 months	TIA	Bilateral	(-)	
7		Female	9	8	TIA	Bilateral	(-)	
8		Female	34	32	CI	Bilateral	(-)	
9		Female	48	44	CI	Bilateral	(-)	
10		Male	47	47	CI	Bilateral	(-)	
11		Female	24	24	headache	Unilateral	(-)	
12		Male	41	40	CI	Bilateral	(-)	
13		Female	22	19	hemiparesis	Unilateral	(-)	
14		Female	19	19	CI	Unilateral	(-)	
15		Female	33	33	CI	Bilateral	(-)	
16		Male	36	36	CI	Bilateral	(-)	
17		Female	39	38	CI	Bilateral	(-)	
18		Female	23	23	dystonia	Bilateral	(-)	

CI, cerebral infarction; TIA, transient cerebral ischemia

*This patient was reported in our previous paper.

### Genetic analysis

Genomic DNA was extracted from peripheral blood using the QIAamp DNA Blood Mini Kit (Qiagen, Hilden, Germany). Direct sequencing was performed on 69 coding exons of *RNF213* using previously described primers [4]. The amino acid coding was based on AB537889. Haplotype analysis was performed using the microsatellite markers flanking the *RNF213* p.V4146A locus (D17S944, D17S949, D17S785, D17S784, and D17S928). The markers were genotyped using ABI Prism Linkage Mapping Set (Version 2; Applied Biosystems, Foster City, CA, USA).

### Cloning

**Fig 1:**
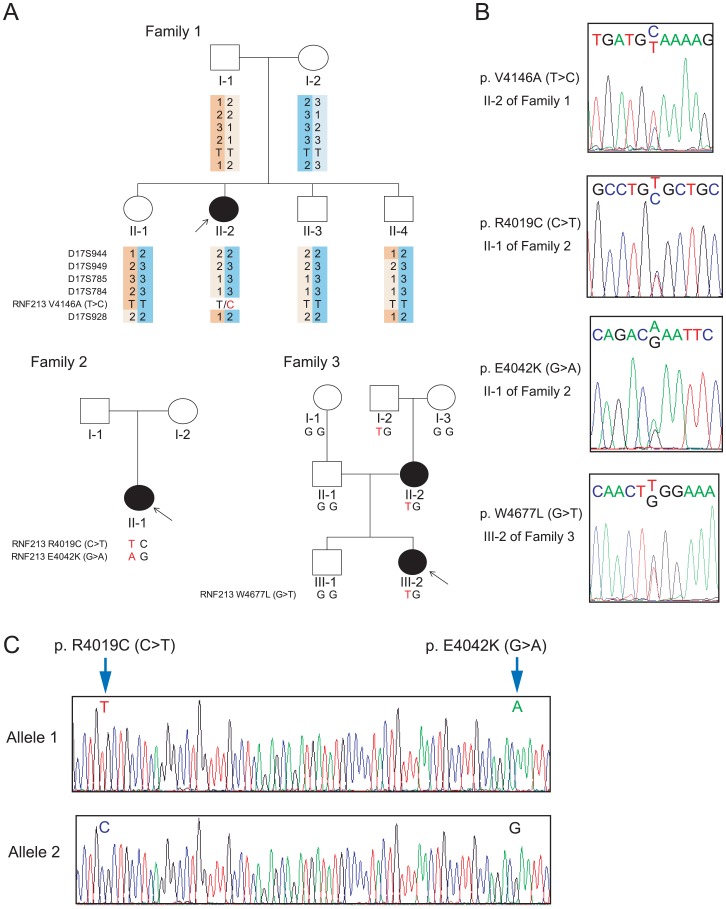
Identification of *RNF213* rare variants in three families. (A) Pedigree chart and genotypes of *RNF213* rare variants and microsatellite markers of the three families. Filled and unfilled symbols indicate affected and unaffected individuals, respectively. Squares and circles represent males and females, respectively. Arrows indicate index case. (B) Sequence chromatography of the identified *RNF213* rare variants. (C) Haplotype for p.R4019C and p.E4042K determined by cloning in II-1 in Family 2.

Cloning of the *RNF213* exon 43 and 44, including *RNF213* p.R4109C and p.E4042K in II-1 in Family 2 (Fig 1A), was performed to determine the haplotype of the two variants. Genomic DNA polymerase chain reaction (PCR) was performed using the following primers; ex43F: 5′-TTG GCC CTG AAT GTG GTG CT-3′, ex44R: 5′-TTC TCT GAG GTC AGG TTT TCT ACC-3′. The PCR product was cloned using the TOPO PCR cloning system (Invitrogen, Carlsbad, CA, USA). Randomly selected colonies were prepared for sequencing.

### Database search for candidate variants

Minor allele frequency (MAF) of variants in the European population was investigated using two variant databases: the 1000 Genomes Project (http://www.1000genomes.org/) and the Exome Variant Server (http://evs.gs.washington.edu/EVS/). The effect of the variants on protein function was assayed using two prediction algorithms: Polyphen2 (http:/genetics.bwh.harvard.edu/pph2) and SIFT (http://sift.bii.a-star.edu.sg/). Variant homology was determined using the protein BLAST search engine (http://blast.ncbi.nlm.nih.gov/Blast.cgi).

### *RNF213* mutant plasmids

*RNF213* mutant plasmids were produced by mutagenesis with the *RNF213* WT plasmid, which was described in our previous study [7]. The p.D4013N, pR4019C or p.V4146A mutation was introduced by PCR-based site-directed mutagenesis using mutated primers (D4013N-F, 5’-CTG TCT GCC CTG CAA CCA CGT GCA CTG C-3’; D4013N-R, 5’-GCA GGC AGT GCA CGT GGT TGC AGG GCA G-3’; R4019C-F, 5’-CGT GCA CTG CCT GTG CTG CCT CAG GGC CTG G-3’; R4019C-R, 5’-CCA GGC CCT GAG GCA GCA CAG GCA GTG CAC G-3’; V4146A-F, 5'- CAG CTT TCA TGA TGC AAA AGA TTA TAT TCA GG-3'; V4146A-R, 5’-CCT GAA TAT AAT CTT TTG CAT CAT GAA AGC TG-3') and Pfu Turbo DNA polymerase (Agilent Technologies, Santa Clara, CA). The generated constructs were confirmed by sequencing.

### Migration and tube formation and assays

Migration and tube formation were assessed as described previously [7]. Briefly, human umbilical vein endothelial cells (HUVECs) (Life Technologies, Carlsbad, CA) were maintained in Medium 200 with low serum growth supplement (Life Technologies). For migration assays, the *RNF213* (WT, D4013N, R4019C and V4146A) plasmids were transfected into 4x10^5^ cells of HUVECs using lipofectamine 3000 (Life Technologies) respectively. The transfected cells were seeded into culture insert (Ibidi, Germany), and after 12 hours, the insert was removed to test cell migration. After 8 h incubation, digital images of wound narrowing (re-endothelialization) were obtained. For tube formation assay, transfected HUVECs (*RNF213* WT, D4013N and V4146A) were seeded onto Geltex LDEV Free matrix (Life Technologies) with μ-slide angiogenesis (Ibidi), and after 15h incubation digital images of the formed tubes were captured. To quantify, the area of re-endothelialization (migration assay) or the area and total length of the tubes, and the number of tube branches (tube formation assay) were calculated using ImageJ software (National Institutes of Health).

### Statistical analysis

Results are presented as mean ± SD. The number of samples is provided in the figure legends. Statistical tests were performed using unpaired Student’s *t*-test. Values of *P* < 0.05 were considered statistically significant.

## Results

### Clinical report

The clinical characteristics of 19 Slovakian and Czech MMD patents (18 probands and one relative) are shown in Table 1. In the majority of the 19 examined MMD patients, clinical manifestations included cerebral infarction (63.1%, 12/19) or transient ischemic attack (15.8%, 3/19), and other clinical symptoms were represented by (hemi)paresis without the development of cerebral infarction (10.5%, 2/19), headache (5.3%, 1/19), or dystonia (5.3%, 1/19). This was consistent with the most common MMD manifestations. In the present study, no patients suffered from other possible manifestations of MMD, such as intracranial hemorrhage, epileptic seizures, or cognitive decline. Detailed clinical information from four patients with *RNF213* rare variants identified in the present study (Fig 1A) are described below.

#### II-2 in Family 1 (Fig 1A)

**Fig 2:**
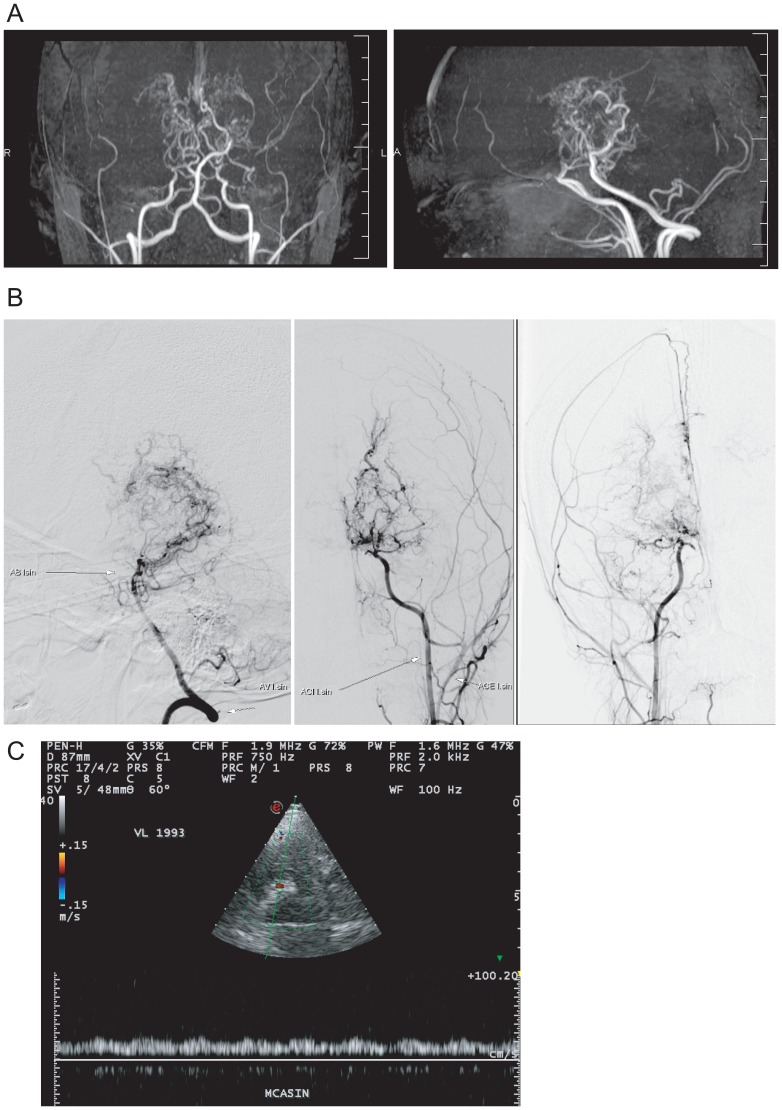
Imaging data of II-2 in Family 1. (A) MRA image. TOF-3D MRA verifies typical steno-occlusive changes of the circle of Willis. Distal T segments of both internal carotid arteries are occluded and basal moyamoya vessels are clearly seen (anteroposterior view, left panel). Typical “puff-of-smoke” look of moyamoya vessels. Internal carotid arteries are relatively hypoplastic compared with the vertebrobasilar system (lateral view, right panel). (B) Digital subtraction angiography. Catheterization angiography of left vertebral artery (left panel), left carotid artery (middle panel), and right carotid artery (right panel). (C) Transcranial color-coded sonography. Severely dampened flow in the M1 segment of the left middle cerebral artery.

A 21-year-old white female was admitted complaining of a 2-day history of headache, visual problems, and right-hand clumsiness in March 2015. Neurological examination disclosed the presence of severe anomic aphasia, agraphia, semantic memory deficits, right-sided hemianopsia, and visual alexia. Initial native brain computed tomography (CT) showed acute cerebral infarction in the left parietal lobe (S1 Fig). Acute cerebral ischemia in the left parieto-occipital region was confirmed by diffusion-weighted imaging magnetic resonance imaging (DWI-MRI) (S2 Fig). Magnetic resonance imaging including magnetic resonance angiography (MRA) revealed severe steno-occlusive changes in the circle of Willis and typical basal moyamoya vessels (Fig 2A, S3 Fig). Digital subtraction angiography (DSA) was used to confirm pathological moyamoya vessels (Fig 2B).

Duplex ultrasound revealed thickening of the carotid bulb in both internal carotid arteries (ICAs) (S4 Fig). Transcranial color-coded sonography (TCCS) confirmed severe flow reduction in the intracranial arteries, especially in the left middle cerebral artery (Fig 2C). We did not find any other laboratory, systemic, or vascular abnormalities, which included diagnosis by CT angiography of the thoracic and abdominal aorta, craniocervical, pulmonary, renal arteries, aorta-iliac bifurcation, iliac arteries, and the arterial system of the lower extremities. Examination of the cerebrospinal fluid (CSF) was completely normal. Echocardiography disclosed a small prolapse with insignificant regurgitation of the anterior leaflet of the mitral valve. Because of the initial suspicion of extracranial and intracranial arteritis, combined immunosuppressive therapy (60 mg prednisone and 100 mg azathioprine daily) was initiated and continued for almost 3 months. Since June 2015, combined antiplatelet therapy (100 mg acetylsalicylic acid and 75 mg clopidogrel daily) was initiated and continues to present. Severe reading difficulties persist, which makes it impossible for the patient to continue with university studies. The patient also does not tolerate severe physical activity or cold weather, which provoke episodes of sudden weakness and the tendency to collapse. Repeated MRI performed in May 2015 showed partial dissolution of the previously present DWI positivity, although new small hyperintense lesions appeared in the parieto-occipital region (S5 Fig). Perfusion CT performed in June 2015 demonstrated a relative cortico-subcortical hypoperfusion with decreased cerebral blood flow, prolonged mean transit time, and time-to-drain values with normalization of cerebral blood volume, as well as postmalatic lesions in the left parietal and occipital lobes (S6 Fig). Revascularization surgery was planned for the left hemisphere.

When analyzing the patient’s previous medical history, we discovered repeated collapses and short periods of muscle tone loss (“drop attacks”), which were initially reported at 9 years of age. These episodes were provoked by various stimuli (physical activity, cold, and vegetative discomforts) and were interpreted as epileptic seizures. At 14 years of age, the patient was admitted to the hospital and MRI was performed. The MRI/MRA images revealed typical findings suggestive of MMD (S7 Fig). However, she was unfortunately not diagnosed with MMD.

Repeated electroencephalograms confirmed epileptiform changes provoked by hyperventilation and photostimulation. Electromyography verified positivity for neurogenic tetania. Valproate was introduced to the treatment strategy. Headache episodes have been mentioned in subsequent years. In June 2014, the patient was admitted to the hospital owing to a sudden onset of fever accompanied by headache and photophobia. Brain CT and CSF examination were interpreted as normal. Laboratory tests and serological findings were negative, and echocardiography and abdominal sonography showed normal results.

The patient has three healthy siblings, and both parents are healthy. We performed complex MRI/MRA and sonographic examinations, including duplex sonography and TCCS in all family members without any definite pathological findings.

#### II-1 in Family 2 (Fig 1A)

**Fig 3:**
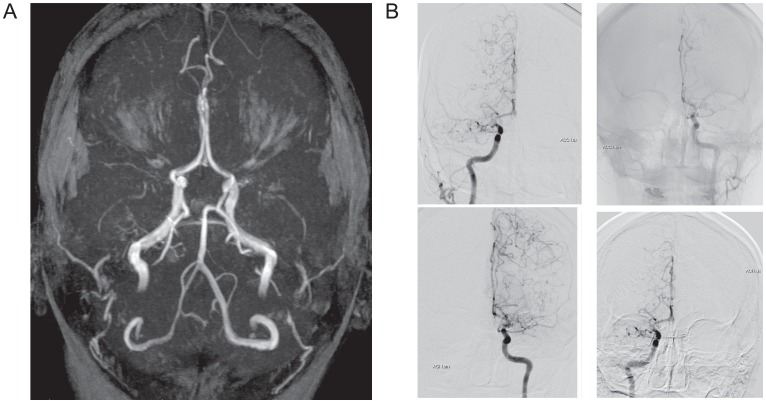
Imaging data of II-1 in Family 2. (A) MRA image at 2015. TOF-3D MRA clearly reveals bilateral occlusion of internal carotid arteries and moyamoya vessel formation. (B) DSA image from 2015.

A white female patient suffered from an acute ischemic stroke in the right hemisphere presenting with a severe left-sided hemiparesis at the age of 19 years (S8 Fig). After intensive care and rehabilitation, she is able to walk independently, despite residual spastic hemiparesis on the left side. Because the patient was trying to plan to get pregnant, she underwent a clinical control examination in May, 2015 at the age of 28 years. The MRA (Fig 3A), DSA (Fig 3B), MRI (S8 Fig), and computed tomography angiography (S9 Fig) were performed, and patient was diagnosed with MMD.

#### II-2 in Family 3 (Fig 1A)

A white female suffered from repeated stroke and underwent extra-intracranial (EC-IC) bypass with encephalo-duro-angio-myo-synangiosis (EDAMS) on the left side at 31 years of age. She also had a history of arterial hypertension, idiopathic thrombocytopenic purpura, sideropenic anemia, and mild hypercholesterolemia. Repeated percutaneous transluminal angioplasty was performed in both renal arteries, the right subclavian artery, and the mesenteric superior artery between the ages of 38 and 43 years. Cerebral angiography, which was performed at 40 years of age, showed left ICA occlusion with collateral blood flow through the left EC-IC bypass, the anterior communicating artery, and the left posterior communicating artery, as well as stenosis of the right ICA (in the siphon) and aneurysmatic dilation of the top of basilar artery (without indication for the endovascular procedure, because of involvement of both posterior cerebral arteries and both superior cerebellar arteries). Additionally, mild post-ischemic changes in the right paraventricular localization were found on brain MRI, which was performed at the same age. One year later, ultrasonographic examination revealed collateral blood flow to the left cerebral hemisphere and the left ophthalmic artery, as well as severe stenosis in the renal arteries. The patient only suffered from intermittent headaches during the follow-up period, and her current neurological status is stable.

#### III-2 in Family 3

A white female, the daughter of the II-2 case, developed severe paresis of the left lower limb with gradual regression at the age of 5 years (April 1994). Identical symptoms occurred also in June 1994, and brain CT revealed narrower lateral ventricles. Although she was treated with immunosuppressants (prednisone and azathioprine), repeated paresis occurred during treatment. The patient was diagnosed with arterial hypertension. In December 1994, angiography was performed with normal findings in the renal arteries but resulted in a MMD diagnosis. In March 1995, the patient underwent EDAMS surgery on both sides. In addition to anti-hypertensive medication, anti-platelet therapy was administered. In May 1996, carotid angiography showed a normal post-operative finding. In March 1997, brain CT revealed mild asymmetry of the lateral ventricles; echocardiography revealed mild septal hypertrophy. In January 2000, MRA was performed with normal findings. Depending on the actual blood pressure values, the anti-hypertensive therapy was altered accordingly. In June 2006, ultrasonographic examination verified MMD (with hypoperfusion in both ICAs, internalization of perfusion in both external carotid arteries); echocardiography showed normal findings. Doppler examination of renal arteries was repeated in December 2007 with normal findings. The patient only suffered from intermittent headaches during the follow-up period; otherwise her current neurological status remains stable. History of Idiopathic thrombocytopenic purpura was absent.

### Identification of *RNF213* rare variants

Exon sequencing of *RNF213* was performed in 18 probands and one relative with MMD. RNF213 exonic variants found in these MMD patients were shown in S1 Table. From these variants, we focus on rare variants whose MAFs in European general population are less than or equal to 1.5% (S1 Table), because MAF of an East Asian MMD-associated variant, p.R4810K was approximately 1.5% [4]. As a result, in addition to p.D4013N, which was previously reported by our group [4], four rare variants were identified in four patients (Table 1, Fig 1A and 1B). A novel variant, *RNF213* p.V4146A (T>C), was identified in II-2 of Family 1. However, direct sequencing in the healthy parents, one sister, and two brothers (I-1, I-2, II-1, II-3, and II-4 in Family 1) revealed the wild-type genotype of p.V4146A, and haplotype mapping using microsatellite genetic markers around p.V4146A confirmed parentages and siblingship. Taken together, these data indicate that the mutation is a *de novo* mutation. II-1 from Family 2 harbored two *RNF213* rare variants, including p.R4019C (C>T), which was previously found in white MMD patients [8], and the novel p.E4042K (G>A) variant. Haplotype analysis by cloning showed that these two mutations occurred on a single allele (Fig 1C). In Family 3, p.W4677L (G>T) was found in proband (III-2) and the affected mother (II-2). Genotyping of p.W4677L in five unaffected (I-1, I-2, I-3, II-1, and III-1) members showed segregation, with exception of I-2.

MAFs of p.V4146A, p.R4019C, and p.E4042K were very rare (< 0.1%), and MAFs of p.W4677L were 1.49% and 1.86% in two European variant databases (Table 2). P.V4146A, p.R4019C, and p.W4677L were predicted to be Probably Damaging/Damaging, while p.E4042K was predicted to be Benign/Tolerated by PolyPhen-2/SIFT, respectively (Table 2). A homology search indicated conservation of valine at position 4146 and tryptophan at position 4677 of *RNF213* in mammals (Table 3). Arginine at position 4019 was conserved among mammals, with exception of rodents (Table 3). Conservation of glutamate at position 4042 was observed in primates (Table 3).

**Table 2:** MAF in database and prediction of functional change of identified RNF213 variants.

Variant	rs Number (dbSNP146)	MAF (%) in European Variants Database		Prediction of Functional Change		Citation
		1000 genome EUR	Exome Variant Server European American	Polyphen 2	SIFT	
p.D4013N	rs397514563	0	0	Possibly damaging	Tolerated	[4, 8]*
p.V4146A	(-)	0	0	Probably damaging	Damaging	
p.R4019C	rs139265462	0	0.09	Probably damaging	Damaging	[8]*
p.E4042K	(-)	0	0	Benign	Tolerated	
p.W4677L	rs61741961	1.49	1.86	Probably damaging	Damaging	

MAF, minor allele frequency

*Previously found in MMD patients

**Table 3:** Homology of identified RNF213 variants.

Species	Amino Acid sequence																					
	p.V4146A											p.R4019C										
*Homo sapiens*	Y	S	F	H	D	**V**	K	D	Y	I	Q	H	V	H	C	L	**R**	C	L	R	A	W
*Pan troglodytes*	Y	S	F	H	D	**V**	K	D	Y	I	Q	H	V	H	C	L	**R**	C	L	R	A	W
*Gorilla gorilla gorilla*	Y	S	F	H	D	**V**	K	D	Y	I	Q	H	V	H	C	L	**R**	C	L	R	A	W
*Pongo abelii*	Y	S	F	N	D	**V**	K	D	Y	I	Q	H	V	H	C	L	**R**	C	L	R	A	W
*Bos taurus*	Y	S	F	H	D	**V**	K	E	Y	I	Q	H	I	F	C	L	**R**	C	I	E	V	H
*Ovis aries*	Y	S	F	C	D	**V**	K	E	Y	I	Q	H	V	F	C	L	**R**	C	I	Q	V	N
*Rattus norvegicus*	Y	S	F	H	E	**V**	K	G	Y	I	Q	H	V	Y	C	L	P	C	I	Q	T	W
*Mus musculus*	Y	S	F	H	E	**V**	K	D	Y	I	Q	H	V	Y	C	L	P	C	I	Q	T	W
Species	Amino Acid sequence																					
	p.E4042K											p.W4677L										
*Homo sapiens*	T	A	L	P	D	**E**	F	S	P	A	V	E	M	R	N	N	**W**	E	K	E	I	A
*Pan troglodytes*	T	A	L	P	D	**E**	F	S	P	A	V	E	M	R	N	N	**W**	E	K	E	I	A
*Gorilla gorilla gorilla*	T	A	L	P	D	**E**	F	S	P	A	V	E	M	R	N	N	**W**	E	K	E	I	A
*Pongo abelii*	T	A	L	P	D	**E**	F	S	P	A	V	E	M	R	N	N	**W**	E	K	E	I	A
*Bos taurus*	T	N	L	P	N	T	F	S	P	T	V	E	E	R	N	R	**W**	E	K	L	V	E
*Ovis aries*	T	D	L	P	D	R	Y	S	P	T	V	E	E	R	N	R	**W**	E	K	L	V	E
*Rattus norvegicus*	T	A	L	P	D	**E**	F	S	P	T	A	G	C	R	N	N	**W**	E	K	H	F	G
*Mus musculus*	T	D	L	P	D	K	F	S	P	T	V	G	C	R	N	N	**W**	E	K	H	F	E

### Evaluation of the effects of *RNF213* p.D4013N, p.R4019C and p.V4146A variants on angiogenic activity of vascular endothelial cells

**Fig 4:**
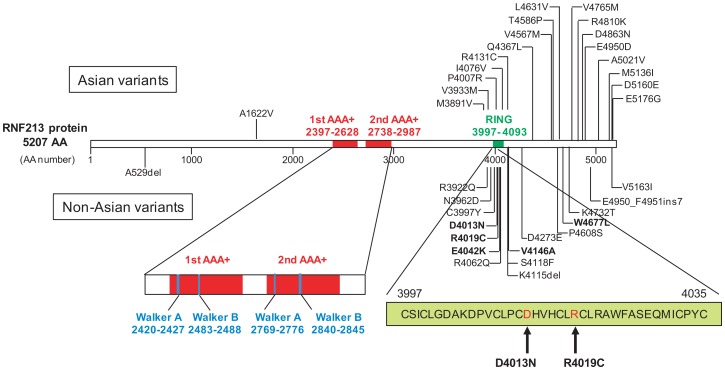
Schematic diagram of *RNF213* rare variants identified in MMD patients. Variants in Asian and white patients are shown above and below the protein, respectively. The five variants identified in MMD patients from this study are shown in bold characters. AA, amino acid; AAA+, ATPase associated with diverse cellular activities domain; RING, RING-finger domain. This figure was modified from the original version described in Reference 6.

**Fig 5:**
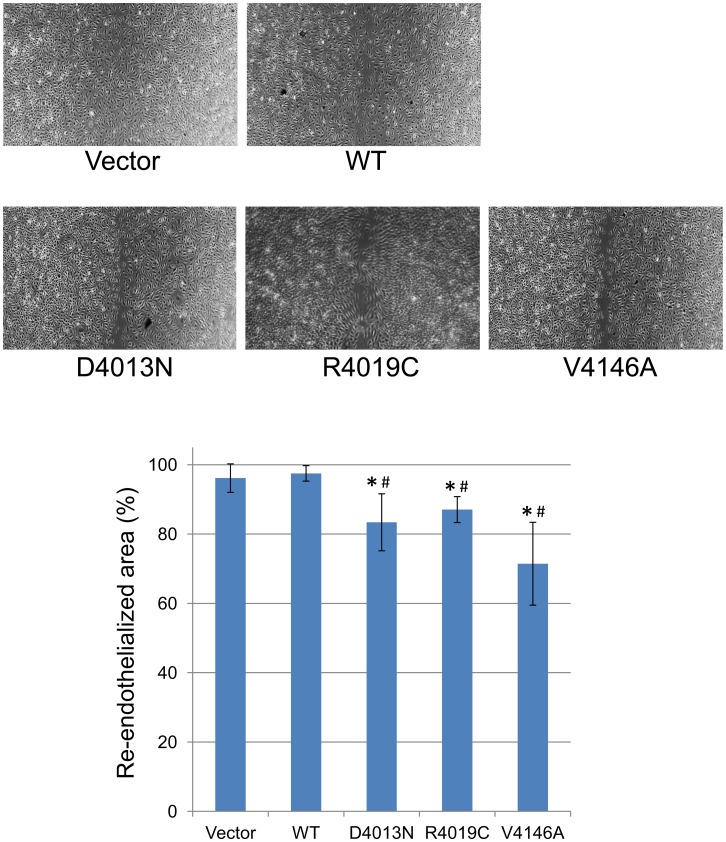
Migration assay using HUVECs transfected with *RNF213* D4013N, R4019C and V4146A. Representative images are shown in upper panel. The re-endothelialized areas were quantified by imaging analysis (lower panel). “Vector” represents backbone vector, not including *RNF213*. Data with bars represent mean ± SD (*n* = 3 or 4). *P < 0.05 compared with vector, #*P* < 0.05 compared with WT according to Student’s *t*-test.

**Fig 6:**
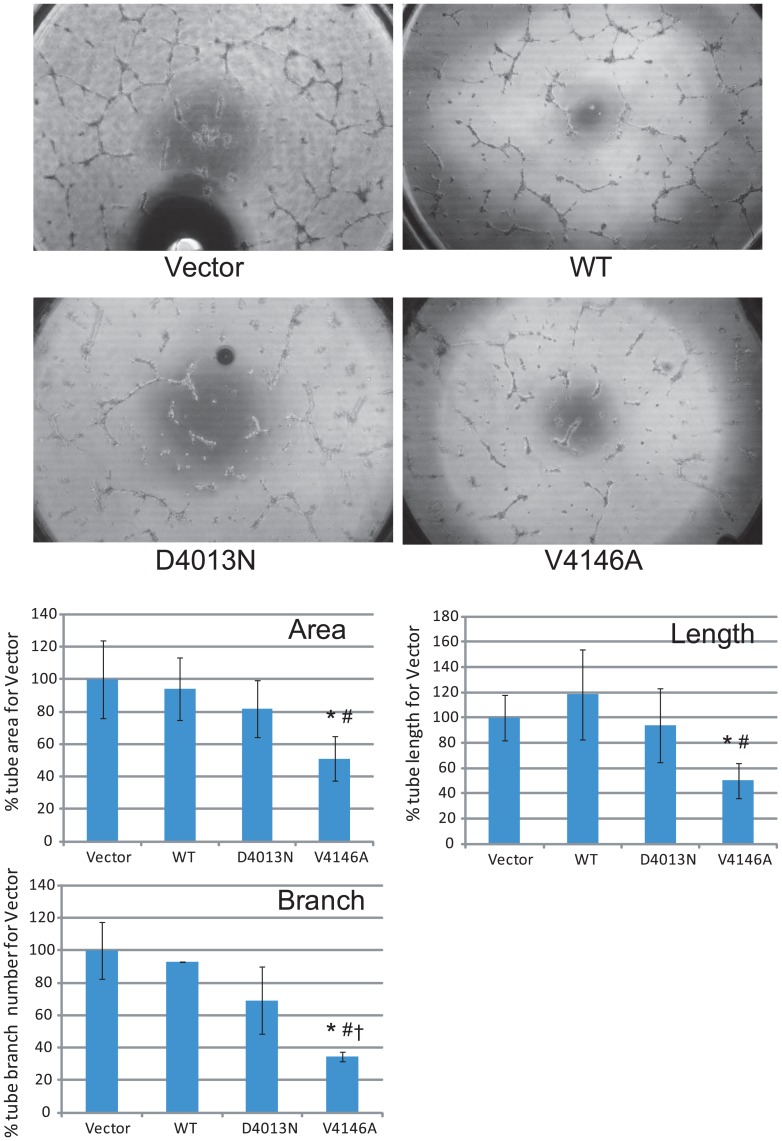
Tube formation assay of HUVECs transfected with *RNF213* D4013N and V4146A. Representative images are shown in upper panel. The tube areas, total tube length, and number of tube branches were quantified by imaging analysis (lower panel). “Vector” represents backbone vector, not including *RNF213*. Data with bars represent mean ± SD (*n* = 3). *P < 0.05 compared with vector, #*P* < 0.05 compared with WT, †*P* < 0.05 compared with D4013N according to Student’s *t*-test.

Among the rare variants found in Slovakian and Czech MMD patients (Table 1), p.D4013N, p.R4019C and p.V4146A are interesting disease-causing variants, because p.D4013N and p.R4019C was previously identified in white familial MMD cases by our and other groups [4,8] and p.V4146A is a *de novo* mutation. Furthermore, both pD4013N and p.R4019C are located in the RING finger domain, which binds E2 and their substrate to function as E3 ligase, while p.V4146A is outside of this domain (Fig 4). To assess the effect of these variants on angiogenic activity of vascular endothelial cells (ECs), we performed migration and tube formation assays using *RNF213* mutant-transfected HUVECs [7,9]. Both *RNF213* D4013N and V4146A significantly decreased re-endothelialization in the migration assay compared with *RNF213* WT and the control vector (backbone vector not including *RNF213*) (Fig 5). In the tube formation assay, HUVECs transfected with *RNF213* V4146A revealed significantly lower tube area, total tube length, and numbers of tube branches compared with the *RNF213* WT and control vector (Fig 6). *RNF213* D4013N tended to reduce angiogenesis, although significant differences were not observed (Fig 6). Migration assay represents capability of cellular migration, and tube formation assay represents the net capability of cell migration, proliferation and survival [10]. While *RNF213* D4013N has a significant inhibitory effect on migration, it does not have a significant inhibitory effects on tube formation, suggesting that migration assay is more sensitive than tube formation assay. It is reported that inhibition of endothelial NO synthase, which is considered to maintain integrin through NO production, attenuated EC migration *in vitro* [11]. Thus, we postulate that *RNF213* D4013N might predominantly affect EC migration through integrin maintenance by inhibition of NO production. To investigate whether the mutation in the RING finger domain may impair cell migration, we further evaluated migration for HUVECs transfected with *RNF213* R4019C. As expected, it did inhibit migration significantly (Fig 5).

## Discussion

In the present study, we identified four rare variants, p.V4146A, p.R4019C, p.E4042K, and W4677L, in four Slovakian or Czech patients with MMD. P.V4146A was demonstrated to be a *de novo* mutation, which had not been previously reported. The case harboring p.V4146A was the third reported MMD case with a *de novo* mutation; the previous described variants were p.K4115del [8] and p.S4118F [12]. Double mutations, p.R4019C and p.E4042K, were also identified in the present study. Although P.E4042K was novel, p.R4019C was previously identified by screening as a single mutation in white MMD patients [8]. Interestingly, these two mutations were inherited on the same allele, suggesting that p.E4042K in the present case was likely a recurrent mutation. Functional predictions (Polyphen 2 and SIFT) showed that p.R4019C was more deleterious than p.E4042K. These results suggest that p.R4019C might play an essential role in MMD onset compared to p.E4042K as previously reported in a white MMD patient [8]. Another variant, p.W4677L was detected in two patients and one non-affected member in the pedigree. Furthermore, this is a rare single nucleotide polymorphism (SNP) (MAF; 1.49–1.86%) in the general European population. These observations indicated relatively low penetrance of p.W4677L. Two recent reports demonstrated that interferons overproduced under inflammatory conditions, such as infection and autoimmune disorders, induced highly up-regulated *RNF213* in ECs [7,13]. Further experimental and epidemiological studies that focus on the link between infectious and autoimmune disease and *RNF213* variants with low penetrance such as p.W4677L are needed.

Functional analysis of p.D4013N, which was previously identified in a Czech MMD family [4], p.R4019C and p.V4146A revealed that these *RNF213* variants induced inhibited angiogenesis in ECs. This lower angiogenesis phenotype was reported to be caused by *RNF213* p.R4810K [7,9], indicating that p.D4013N, p.R4019C and p.V4146A are the likely causative mutations for MMD. The present study is the first to reveal the inhibitory effects of non-p.R4810K *RNF213* variants on angiogenesis. Furthermore, these results strongly support the concept that reduced angiogenesis plays an important role in MMD etiology.

The RNF213 protein harbors two AAA+ and one RING-finger domain, which have been demonstrated to exhibit ATPase and E3 activity, respectively [4,14]. The *RNF213* rare variants identified in the present study were located on the locus corresponding to the region from the RING-finger domain to the C-terminus of the RNF213 protein. This was consistent with characteristics previously described in *RNF213* rare variants in Asian and white MMD patients (Fig 4) [6]. It should be noted that p.D4013N, p.R4019C, and E4042K are located in the RING-finger domain, and these mutations could alter angiogenic activity through E3 ligase activity. In the present study, we found that mutations (p.D4013N and p.R4019C) in this domain decreased re-endothelialization, which is associated with integrin homeostasis and NO production [11]. We postulated that effects of mutations in the RING finger domain on net angiogenesis are milder than p.V4146 or p.R4810K [7] because those mutations could be recoverable in the tube formation. Further study is needed to confirm our finding.

Genetic epidemiological studies have demonstrated that *RNF213* p.R4810K is frequently observed in East Asian MMD patients (Japan and Korea, 80–90%; China, around 20%) and was demonstrated to have a strong association with the disease [6]. Our present screening for *RNF213* revealed non-R4810K *RNF213* rare variants in 22.2% (4/18 probands) of Slovakian or Czech MMD probands (Table 1), raising the possibility that *RNF213*-associated MMD may be present in a relatively large population of non-Asian MMD cases. This concept was supported by a recent report showing a relatively high frequency of non-R4810K *RNF213* rare variants (10.6% (10/94 probands)) in non-Asian MMD patients from North America [8].

## Conclusions

The present study suggests that *RNF213* may also be a major causative gene in a relative large population of white patients. Routine screening should be performed for *RNF213* rare variants in MMD patients regardless of ethnic background. This could be useful for a definitive MMD diagnosis and also serve to provide a better understanding of MMD etiology.

## Supporting Information

S1 Fig Native brain computed tomography of II-2 in Family 1. (DOCX)

S2 Fig MRI imaging (diffusion-weighted imaging, DWI) of II-2 in Family 1. (DOCX)

S3 Fig MRI imaging (T2-weighted) of II-2 in Family 1. (DOCX)

S4 Fig Duplex ultrasound image of II-2 in Family 1. (DOCX)

S5 Fig Repeated MRI imaging (DWI) of II-2 in Family 1. (DOCX)

S6 Fig Perfusion-computed tomography of II-2 in Family 1. (DOCX)

S7 Fig MRI imaging (T1-weighted image) of II-2 in Family 1 coronal scan. (DOCX)

S8 Fig MRI imaging (FLAIR) of II-1 in Family 2 from 2009 and 2015. (DOCX)

S9 Fig CT angiography of II-1 in Family 2 from 2015. (DOCX)

S1 Table RNF213 exonic varinats found in total 19 Slovakian or Czech MMD patients. (DOCX)
