# Supplementary material for: RNF213 Rare Variants in Slovakian and Czech Moyamoya Disease Patients
Source: PLoS One. 2016 Oct 13;11(10):e0164759. doi: 10.1371/journal.pone.0164759 (PMC5063318; doi:10.1371/journal.pone.0164759)
Supplement: S2 Fig — (DOCX) [file pone.0164759.s002.docx]

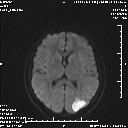

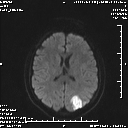

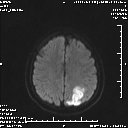

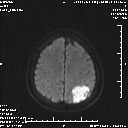

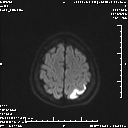


**S2 Fig. MRI imaging (diffusion-weighted imaging, DWI) of II-2 in Family 1.**

*axial scans:* acute ischemic infarction in the parieto-occipital region.
