# Supplementary material for: RNF213 Rare Variants in Slovakian and Czech Moyamoya Disease Patients
Source: PLoS One. 2016 Oct 13;11(10):e0164759. doi: 10.1371/journal.pone.0164759 (PMC5063318; doi:10.1371/journal.pone.0164759)
Supplement: S3 Fig — (DOCX) [file pone.0164759.s003.docx]

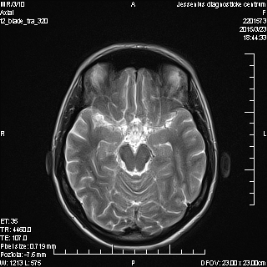


**S3 Fig. MRI imaging (T2-weighted) of II-2 in Family 1.**

*axial scan at the basal cistern level:* The horizontal portion of the middle cerebral artery is not identified. Small characteristic signal voids suggesting moyamoya vessels can be seen.
