# Supplementary material for: RNF213 Rare Variants in Slovakian and Czech Moyamoya Disease Patients
Source: PLoS One. 2016 Oct 13;11(10):e0164759. doi: 10.1371/journal.pone.0164759 (PMC5063318; doi:10.1371/journal.pone.0164759)
Supplement: S4 Fig — (DOCX) [file pone.0164759.s004.docx]

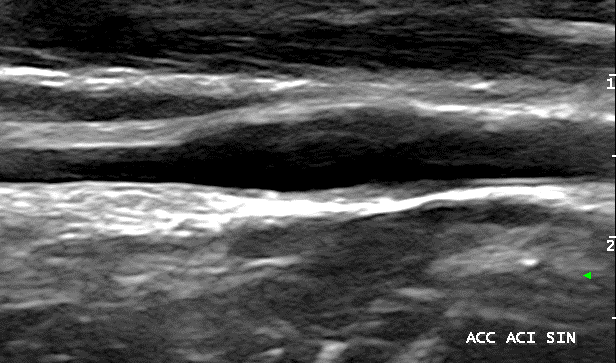

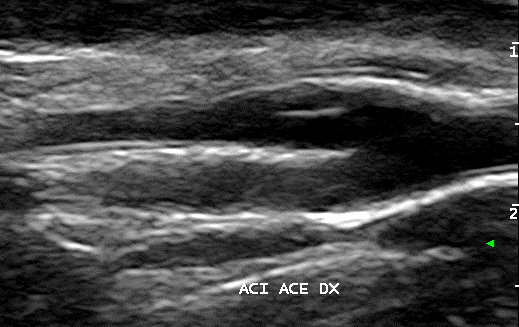


**S4 Fig. Duplex ultrasound image of II-2 in Family 1.**

Left common and internal carotid arteries show slight thickening of the carotid bulb (upper panel) and right common, internal, and external carotid arteries (lower panel).
