# Supplementary material for: RNF213 Rare Variants in Slovakian and Czech Moyamoya Disease Patients
Source: PLoS One. 2016 Oct 13;11(10):e0164759. doi: 10.1371/journal.pone.0164759 (PMC5063318; doi:10.1371/journal.pone.0164759)
Supplement: S5 Fig — (DOCX) [file pone.0164759.s005.docx]

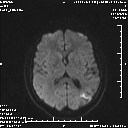

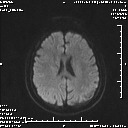


**S5 Fig. Repeated MRI imaging (DWI) of II-2 in Family 1.**

*axial scans:* new small hyperintense lesion in the left parieto-occipital region (left panel). Regression of DWI positivity in the left parietal region compared with S2 Fig (right panel).
