# Supplementary material for: RNF213 Rare Variants in Slovakian and Czech Moyamoya Disease Patients
Source: PLoS One. 2016 Oct 13;11(10):e0164759. doi: 10.1371/journal.pone.0164759 (PMC5063318; doi:10.1371/journal.pone.0164759)
Supplement: S6 Fig — (DOCX) [file pone.0164759.s006.docx]

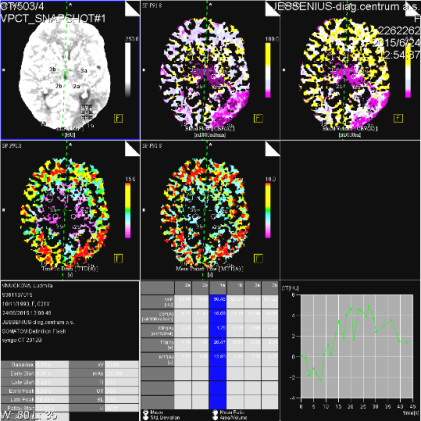


**S6 Fig. Perfusion-computed tomography of II-2 in Family 1.**

Normal cerebral blood flow (CBF) values in central and deep brain areas (CBF up to 63 mL/100 mL/min), as well in corona radiata; lower CBF values (CBF up to 20 mL/100 mL/min), prolonged mean transit time (MTT = 10 s), prolonged time to drain (TTD = 15 sec), and normal cerebral blood volume (CBV = 2 mL/100 g) in cortico-subcortical areas; postmalatic lesion in the left parieto-occipital region.
