# Supplementary material for: RNF213 Rare Variants in Slovakian and Czech Moyamoya Disease Patients
Source: PLoS One. 2016 Oct 13;11(10):e0164759. doi: 10.1371/journal.pone.0164759 (PMC5063318; doi:10.1371/journal.pone.0164759)
Supplement: S7 Fig — (DOCX) [file pone.0164759.s007.docx]

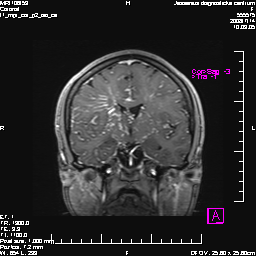


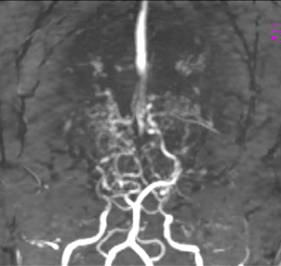


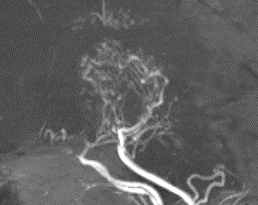


**S7 Fig. MRI imaging (T1-weighted image) of II-2 in Family 1 coronal scan.**

Bizarre vasculature in the right hemisphere compared with absence of vascular bed signals in the left hemisphere (upper panel). Magnetic resonance angiography: moyamoya vessels at the circle of Willis level, anteroposterior (middle panel) and lateral (lower panel).
