# Supplementary material for: RNF213 Rare Variants in Slovakian and Czech Moyamoya Disease Patients
Source: PLoS One. 2016 Oct 13;11(10):e0164759. doi: 10.1371/journal.pone.0164759 (PMC5063318; doi:10.1371/journal.pone.0164759)
Supplement: S8 Fig — (DOCX) [file pone.0164759.s008.docx]

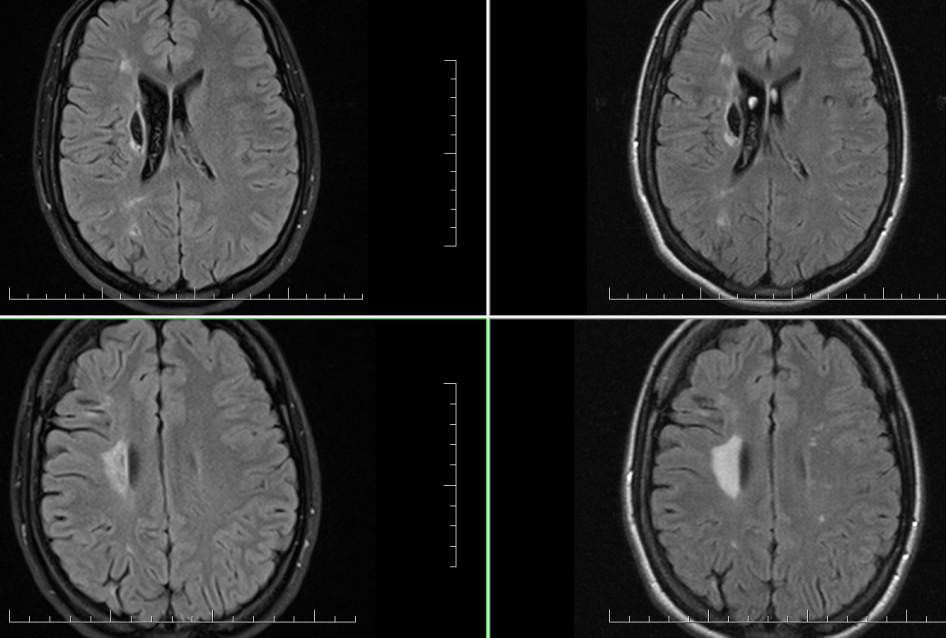


**S8 Fig. MRI imaging (FLAIR) of II-1 in Family 2 from 2009 and 2015.**

FLAIR images from 2009 show capsular and parieto-occipital infarctions in the right hemisphere (left panels), FLAIR images from 2015 show old infarctions in the right hemisphere and new asymptomatic small multiple ischemic lesions in the left hemisphere (right panels).
