# Supplementary figures and images for: RNF213 Rare Variants in Slovakian and Czech Moyamoya Disease Patients
Source: PLoS One. 2016 Oct 13;11(10):e0164759. doi: 10.1371/journal.pone.0164759 (PMC5063318; doi:10.1371/journal.pone.0164759)

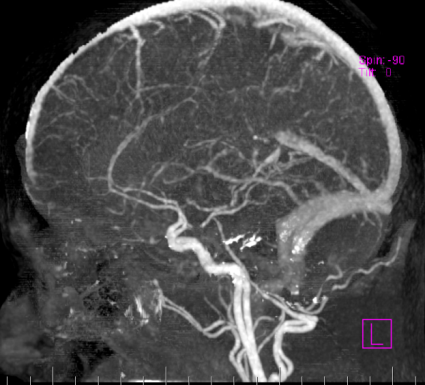


**S9 Fig. CT angiography of II-1 in Family 2 from 2015.**

Supplement: S9 Fig — (DOCX) [file pone.0164759.s009.docx]
