# Supplementary material for: RNF213 Rare Variants in Slovakian and Czech Moyamoya Disease Patients
Source: PLoS One. 2016 Oct 13;11(10):e0164759. doi: 10.1371/journal.pone.0164759 (PMC5063318; doi:10.1371/journal.pone.0164759)
Supplement: S1 Table — (DOCX) [file pone.0164759.s010.docx]

**S1 Table. RNF213 exonic varinats found in total 19 Slovakian or Czech MMD patients.**

| rs Number  (dbSNP146) | MAF (%) in 1000 genome EUR | RNF213 variant | Reference genotype | Proband No. (ID in present paper)* | | | | | | | | | | | | | | | | | | |
| --- | --- | --- | --- | --- | --- | --- | --- | --- | --- | --- | --- | --- | --- | --- | --- | --- | --- | --- | --- | --- | --- | --- |
|  |  |  |  | 1 | 2 (II-2 Family 1) | 3 (II-1 Family 2) | 4 (III-2 Family 3) | mother of 4 (II-2 Family 3) | 5 | 6 | 7 | 8 | 9 | 10 | 11 | 12 | 13 | 14 | 15 | 16 | 17 | 18 |
| rs17853714 | 16.2 | p.P126P | GG |  | GA |  |  |  |  |  |  |  | GA |  | GA |  |  |  |  |  |  |  |
| rs7215243 | 19.9 | p.P151P | AA |  | AG |  |  |  |  |  |  |  | AG |  | AG |  | AG |  | AG |  |  |  |
| rs17857135 | 14.0 | p.M270T | TT |  | TC |  |  |  |  |  |  |  |  |  |  |  |  |  |  |  |  |  |
| rs17853989 | 16.5 | p.M321T | TT |  | TC |  |  |  |  |  |  |  |  |  | TC |  |  |  |  |  |  |  |
| rs17853713 | 16.5 | p.K330T | GG |  | GA |  |  |  |  |  |  |  |  |  | GA |  |  |  |  |  |  |  |
| rs72849841 | 14.0 | p.P729L | CC |  |  |  |  | CT |  |  |  |  |  |  |  |  |  |  |  |  |  |  |
| rs61359568 | 5.7 | p.A1041T | GG |  |  |  |  |  |  |  |  | GA |  |  |  |  |  |  |  |  |  | GA |
| rs9913636 | 49.9 | p.E1272Q | GG | GC | GC |  | GC | CC |  | CC | CC |  | GC |  | CC | GC | CC | GC | GC | GC |  |  |
| rs8074015 | 29.7 | p.D1331G | AA | AG | AG | AG | AG | GG | AG | GG | GG | AG | GG |  | GG | AG | GG | GG | AG | AG |  | AG |
| rs9908287 | 33.1 | p.V1340V | CC | CG | CG |  | CG | GG | CG | GG | GG | CG | GG |  | GG | CG | GG | CG | CG | CG |  | CG |
| rs4890009 | 29.8 | p.A1550A | GG | GA | GA | GA | GA |  | GA |  |  |  |  |  | AA | GA |  |  |  | AA |  | GA |
| rs61600413 | 3.4 | p.C3008R | TT |  |  | TC |  |  |  |  |  |  |  |  |  | TC |  |  |  |  |  |  |
| rs61741791 | 6.4 | p.P3759P | GG |  |  |  |  |  |  |  |  |  |  |  |  |  |  |  |  |  |  | GA |
| rs35332090 | 10.4 | p.V3838L | GG |  |  |  |  |  |  |  |  |  |  |  |  |  |  | GC |  |  |  | GC |
| rs61740658 | 8.8 | p.E3915G | AA |  |  |  |  |  |  |  |  |  |  |  |  |  |  | AG |  |  |  | AG |
| rs397514563 | 0.0 | p.D4013N | GG | GA |  |  |  |  |  |  |  |  |  |  |  |  |  |  |  |  |  |  |
| rs139265462 | 0.0 | p.R4019C | CC |  |  | CT |  |  |  |  |  |  |  |  |  |  |  |  |  |  |  |  |
| (-) | 0.0 | p.E4042K | GG |  |  | GA |  |  |  |  |  |  |  |  |  |  |  |  |  |  |  |  |
| (-) | 0.0 | p.V4146A | TT |  | TC |  |  |  |  |  |  |  |  |  |  |  |  |  |  |  |  |  |
| rs116948489 | 6.6 | p.R4269R | GG |  |  |  |  |  |  |  |  |  |  |  |  |  |  |  |  |  |  | GA |
| rs4889848 | 8.9 | p.H4557H | CC | CT | CT |  |  |  |  |  |  |  |  |  |  | CT |  |  |  | CT |  | TC |
| rs61745599 | 8.9 | p.L4649V | CC |  |  |  | CG |  |  |  |  |  |  |  |  |  |  |  |  |  |  |  |
| rs61741961 | 1.5 | p.W4677L | GG |  |  |  | GT | GT |  |  |  |  |  |  |  |  |  |  |  |  |  |  |
| rs3185057 | 9.0 | p.A5107A | GG |  |  |  |  |  |  |  |  |  |  |  |  |  |  | GA |  |  |  | GA |
| rs8072774 | 9.0 | p.V5028I | GG |  |  | GA |  |  |  |  |  |  |  |  |  |  |  |  |  |  |  |  |

*Information of the MMD patients were described in Table 1.

Blank cells in each patients present reference (WT) genotype.
